# Approximately Model of the Maximum Temperature on the Chip Surface

**DOI:** 10.3390/ma14102592

**Published:** 2021-05-16

**Authors:** Marian Bartoszuk

**Affiliations:** Faculty of Mechanical Engineering, Opole University of Technology, 45-271 Opole, Poland; m.bartoszuk@po.edu.pl; Tel.: +48-501-586-712

**Keywords:** orthogonal cutting, approximately model, cutting temperature, chip, thermographic images

## Abstract

This article presents an approximately model that allows for the determination of the maximum temperature of the chip surface in dry orthogonal turning. The mathematical formula describing the maximum temperature of the chip surface was formulated based on experimental data. The experiments were carried out for orthogonal cutting of austenitic steel AISI 321 with flat rake face carbide inserts that were made of tungsten carbide H10F, both uncoated and coated, with coatings of varied arrangement. Thermographic images of the cutting zone were used to verify the correctness of the approximately model. The obtained results show good agreement between the modelling results and experimental studies. The discrepancy of the maximum temperature values of the top surface of the chip does not exceed 6.4%.

## 1. Introduction

Temperature measurements during cutting provide significant information regarding the process itself. In practice, it is impossible to measure the cutting temperature directly. Indirect values must be used for the purpose of its measurement, which are directly connected to the cutting temperature. Frequently, measurements in a specified point of the cutting zone are taken, i.e., inside the cutting tool or on its surface, in order to define the temperature distribution in the area of interest. Recently, a great deal of valuable information regarding the distribution of temperature on the cutting zone surfaces is obtained based on thermal imaging measurements. For example, Rech [1], in his investigations on the orthogonal turning of AISI 4142H steel, used a CCD-IR camera that was pointed towards the lateral area of a disc and the tool edge. The temperature distribution was analysed in the cutting tool and the workpiece material. Abukhshim et al. [2] employed a similar solution. In their research, they specified the temperature distribution for the whole cutting zone during the orthogonal cutting of AISI 4140 steel with uncoated carbide inserts. Thermographic measurements during cutting were also used by Jaspers and Dautzenberg [3,4]. They analysed thermographic images of the tool rake face and the chip using an IR camera that was placed directly above the cutting zone. In this research, an innovative method of measurement chain calibration by means of a special vacuum chamber is proposed.

Thermovision images of the cutting zone may be the basis for the verification of the simulation model of cutting with reference to the heat distribution between the chip, the cutting tool, and the workpiece. The temperature in the characteristic points [5] or the maximum value of the temperature on the analysed surface is usually measured to compare the heat distribution values. The top surface of the chip is the region that is often used for comparison. Occasionally, the researchers are unable to use the full range of experimental methods; thus, it would be expedient to approximately determine the distribution of temperature along the chip. However, literature sources do not provide any model enabling the determination of the chip temperature. They also lack a model to calculate the maximum temperature of the top surface of the chip. In view of the presented considerations and inspired by the research of Jaspers et al. [3,4], this study seeks to formulate an equation allowing for the calculation of the maximum temperature of the top surface of the chip.

## 2. Experimental Details

### 2.1. Characterisation of Machining Conditions and the Machining System

This paper focuses on dry orthogonal cutting of austenitic steel AISI 321 with uncoated carbide inserts, as well as inserts with coatings of varied arrangements. Table 1 presents the composition and structure of the coatings. The cutting edges were made of H10F carbide. The experiments were performed with the use of a PTNGR 2020-16 tool holder (PAFANA S.A., Pabianice, Poland) that ensures an orthogonal setup of the TNMA 160,408 cutting insert with a flat rake face. The cutting parameters assumed for the tests are as follows: cutting speed *v_c_* = 100 m/min, feed *f* = 0.20 mm/rev, and depth of cut *a_p_* = 2 mm.

**Figure 1:**
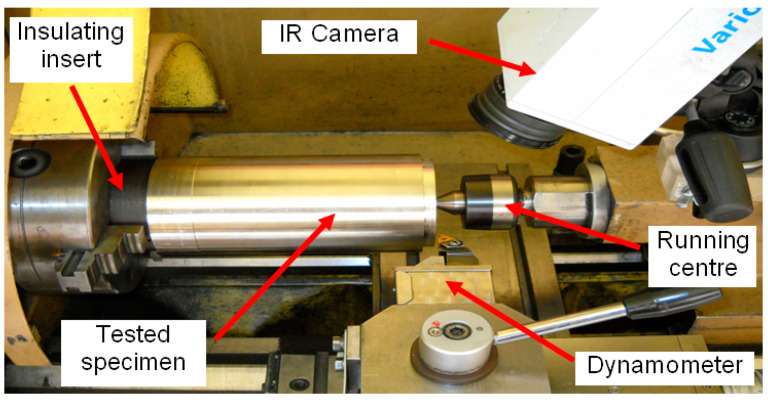
View of the sample in the machining space of the test bench [6].

The cutting was performed at a station made of the lathe TUM-35D1, equipped with a KISTLER 9257B dynamometer (with a KISTLER 5019B amplifier, Kistler Holding AG, Winterthur, Swiss), an original system for measuring the thermoelectrical force generated at the contact area of the tool and the workpiece, and a VarioCAM thermal camera by JENOPTIK (JENOPTIK AG, Jena, Germany) with the IRBIS 3 software (version: 30, InfraTec, Dresden, Germany) for archiving and processing thermographic images. Figure 1 shows the machining space of the test bench. 

### 2.2. Calibration of the Measurement Chain

**Figure 2:**
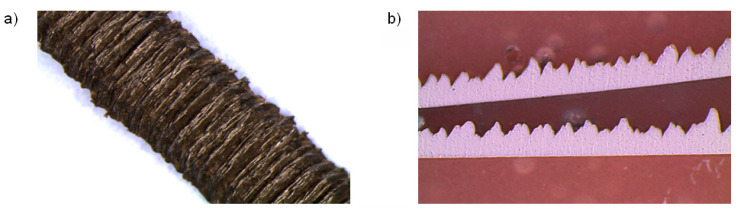
The chip after orthogonal turning with a cutting speed of *v_c_* = 50 m/min and a feed of. *f* = 0.1 mm/rev: (**a**) view of the chip surface at the 10.5× magnification; (**b**) longitudinal section of the chip at the 24× magnification [6].

The chip surface observed in the thermographic images has a complicated and furrowed texture because of the character of the chip formation process (Figure 2). The emissivity of such a surface differs heavily from the emissivity observed for the machined surfaces, as can be seen from the literature data [4,7,8,9]. Usually, the calibration of the thermovision measurement channel is performed based on the black body [8] or specially made surfaces with known emissivity [9]. These can be considered as idealised conditions.

Jaspers [3,4] proposed a different solution, who calibrated the thermovision measurement channel using a chip contained in a special vacuum chamber, heated with the flow of the current. Jaspers [4], similarly to Arrazola et al. [7], demonstrated the minimal influence of oxidation on the change of the emissivity factor. Based on their work, the present research neglects the effect of oxidation of the tested surfaces on the value of the emissivity factor.

**Figure 3:**
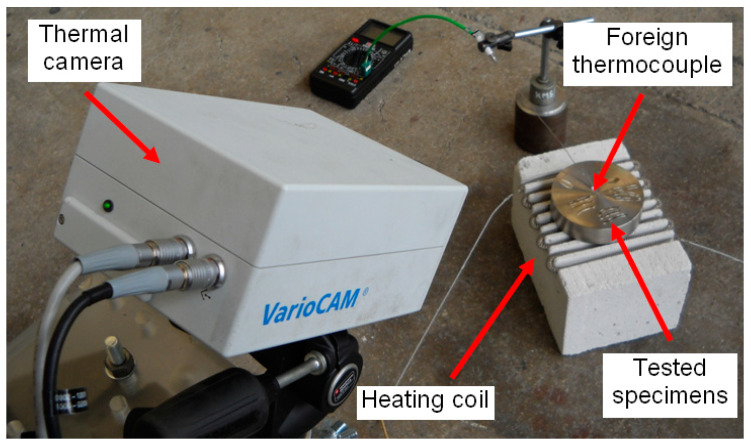
Bench for IR camera calibration [6].

In order to reflect the real conditions during the calibration as closely as possible, the measurement chain was calibrated with the use of chips tat were placed on a plate made of the test steel. A special thermally conductive ceramic glue DRAGON (Wytwórnia Chemiczna DRAGON, Kraków, Poland) was applied to reduce the loss of thermal conduction between the base plate and the chip surface. As in the experimental tests, the surface of the chip directly contacted the surrounding atmosphere during calibration. The bench used for the calibration of the thermovision measurement channel is illustratively presented in Figure 3.

**Figure 4:**
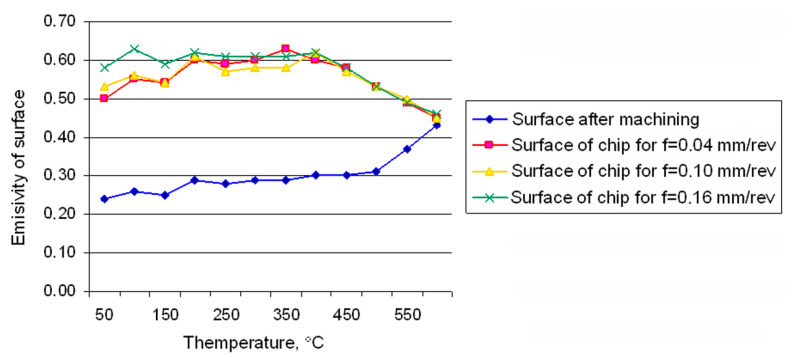
Changes in the emissivity factor of the chip surfaces and AISI 321 after turning versus temperature [6].

The analysis of the fluctuation of the emissivity factor values determined for chips produced at the cutting speed of *v_c_* = 50 m/min. shows that, regardless of the cutting parameters, the observed surfaces of the chips have very similar values of emissivity (Figure 4). The nature of the changes, especially in the range of higher temperatures, is almost identical for all of the analysed cases. At the same time, the performed tests show significant differences in the values of emissivity for chips and the machined surface of the base plate with roughness of about *Ra* = ~1.215 µm. These differences are particularly striking for temperatures below 400 °C. The emissivity for the turned surface in the considered range of temperature was half as much as the emissivity of the surfaces of the chips.

### 2.3. Thermovision Measurement of Heat Distribution in the Cutting Zone

**Figure 5:**
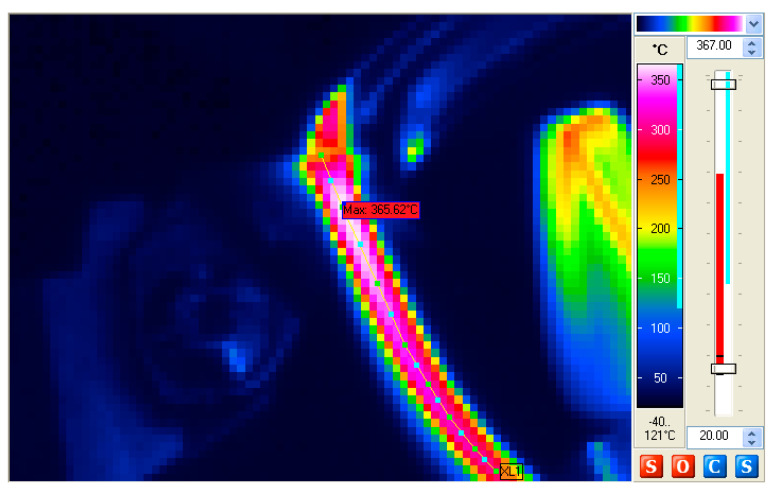
A thermogram of the cutting zone recorded for the insert coated with AlTiN at a cutting speed of *v_c_* = 100 m/min and feed of *f* = 0.20 mm/rev.

During the experiments, the IR camera was positioned directly above the cutting zone. Such a position of the camera allowed for the recording of the temperature distribution in the whole cutting zone. Figure 5 shows an exemplary thermographic image of the cutting zone. Special attention was paid to the maximum temperature of the top surface of the chip as a parameter that may be helpful in verifying the simulation results.

**Figure 6:**
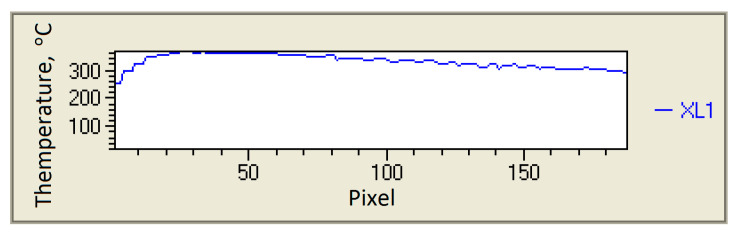
The heat distribution along the centre line of the chip obtained for the insert coated with AlTiN, with cutting parameters of *v_c_* = 100 m/min., *f* = 0.20 mm/rev.

The analysis of the thermograms of the cutting zone was carried out by means of IBRIS 3 software (version: 30, InfraTec, Dresden, Germany), which enabled the automatic finding of the regions of the highest temperature. For comparative purposes, the distribution of the temperature was also determined along the centre line of the chip (Figure 5). Figure 6 shows an exemplary course of changes in the temperature of the top surface of the chip.

## 3. Results and Discussion

### 3.1. Results of Experimental Investigations

From the literature [3,10,11,12] and personal observations, it follows that the maximum temperature of the top surface of the chip is subject to some fluctuations. The maximum temperature changes its value and location with respect to the chip beginning. Similar to the fluctuations of the temperature of the chip surface, but to a much smaller extent, the thickness of the chip and the frequency of segmentation also undergo changes. Therefore, the texture of the surface of the chip changes, which directly affects the change of the emissivity factor. Thus, it may be concluded that the actual temperature fluctuations are much smaller. The change in the emissivity of the test surface mainly causes the measured fluctuations of the maximum temperature of the chip, sometimes reaching 10 or 15%.

**Figure 7:**
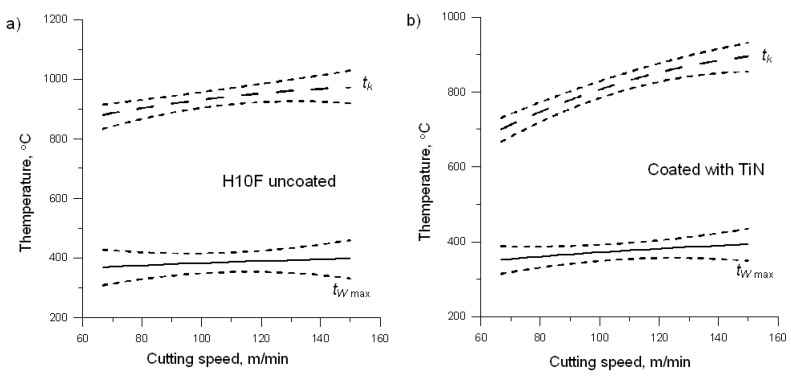

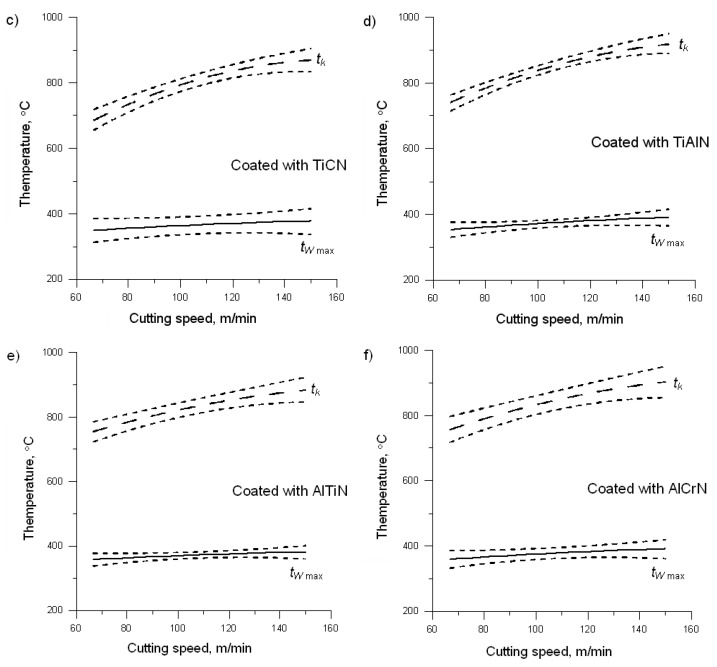
Contact and maximum temperatures of the chip surface for the H10F insert: (**a**) uncoated and coated with (**b**) TiN, (**c**) TiCN, (**d**) TiAlN, (**e**) AlTiN, and (**f**) AlCrN.

Figure 7 presents a graphical comparison of the average temperature of contact and the maximum temperature of the surface of the chip. By comparing both of the values of temperature, it can be noted that, regardless of the tool material, the maximum temperature of the chip surface stabilises at a level that is 50% lower than the average contact temperature. This may be due to the fact that the cooling conditions in all cases were identical. Therefore, you can conclude that the chip temperature will stabilise at the level proportional to the value of the temperature reached during the passage of the workpiece material through the primary and secondary deformation zones.

### 3.2. Approximation Model for Determination of Maximum Temperature on the Chip Surface

A close study of the model formulas of the heat transfer in the cutting zone, including the formulas for determining the heat partition coefficient [13,14,15,16,17], shows that they are mainly based on the thermophysical properties of the tool and workpiece materials, rarely based on the geometrical dimensions of the contact zone, and even more seldom on the cutting parameters. Based on the similar premises, the author undertook to develop a formula that enables to approximately determine the maximum temperature of the chip surface for the orthogonal cutting. Taking literature data into account and based on the experimental data, the author created a formula describing the maximum temperature of the chip surface for the orthogonal cutting of austenitic steel. This relation may be described with the following equation:

(1) $$t_{Wmax}=-400359755\times \left(\frac{e_{c}}{\rho_{W}\times c_{pW}}\right)+568.846\;{}^{\circ}C$$

where: 

*e_c_*—volumetric specific cutting energy J/m^3^, 

*ρ_W_*—workpiece density kg/m^3^, and

*c_pW_*—specific heat capacity of the workpiece J/(kg·°C).

**Figure 8:**
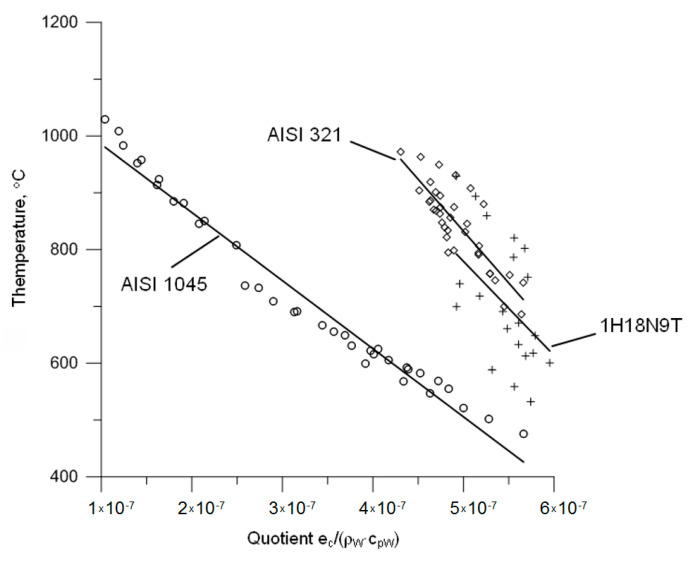
The progress of changes of the average contact temperature versus the quotient *e_c_*/(*ρ_W_·c_pW_*).

The above correlation is only valid for the test AISI 321 austenitic steel. Aiming at the development of a formula that is valid for different metallic materials producing a continuous chip in machining, the effect of the quotient *e_c_*/(*ρ_W_·c_pW_*) on the value of contact temperature was analysed. It appears that more precise information may be provided by analysing this quotient combined with the maximum temperature of the chip surface; however, the author does not have access to an appropriate experimental database. The graph of the contact temperature that is presented in Figure 8 versus the product *e_c_*/(*ρ_W_·c_pW_*) for AISI 321 steel and materials previously investigated by the author suggests a conclusion that all of the investigated materials produced functions of a similar character of changes arranged proportionally to each other.

Following this logic, a general form of an equation describing the maximum temperature of the chip surface for different machined materials may be formulated. This equation was developed empirically based on Equation (1) and the relationships shown in Figure 8. The general notation of this formula is shown below:

(2) $$t_{Wmax}=k_{E}\times \left[A\times \left(\frac{e_{c}}{\rho_{W}\cdot c_{pW}}\right)+B\right]\;{}^{\circ}C$$

where:

*A* and *B*—equation parameters that are determined from approximation,

*k_E_*—proportionality factor.

The proportionality factor *k_E_* entered into Equation (2) introduces a correction connected with the machinability of the tested material. It is established on the basis of Young’s modulus of the test material. Still, it should be noted that:

*k_E_* < 1—for materials with Young’s modulus higher than that of the reference material,

*k_E_* = 1—for the reference material (austenitic steel AISI 321), and

*k_E_* > 1—for materials with Young’s modulus lower than that of the reference material.

The *k_E_* factor is an estimate. Its exact value for any other processed machined material must be determined by making analogous measurements.

The analysis of the notation of Formula (2) for the estimation of the maximum temperature of the surface of the chip t_Wmax_ indicates that the accuracy of such calculations will depend on the accuracy of the estimation of coefficients A and B and, in particular, on the precision with which the proportionality coefficient *k_E_* is adopted. The values of coefficients A and B directly result from the calculations and, respectively, are: *A* = −400359755 and *B* = 568.846. Therefore, it is the proportionality factor *k_E_*, which is experimentally selected according to the machinability of the workpiece material, which is identified as the main source of inaccuracy. The general principles for selecting the value of this factor are shown above. However, the results of the pilot study that are presented in this article do not allow for precise values of the proportionality factor to be determined. A table of suggested *k_E_* values can only be developed in further research conducted for a wide range of workpiece materials.

**Figure 9:**
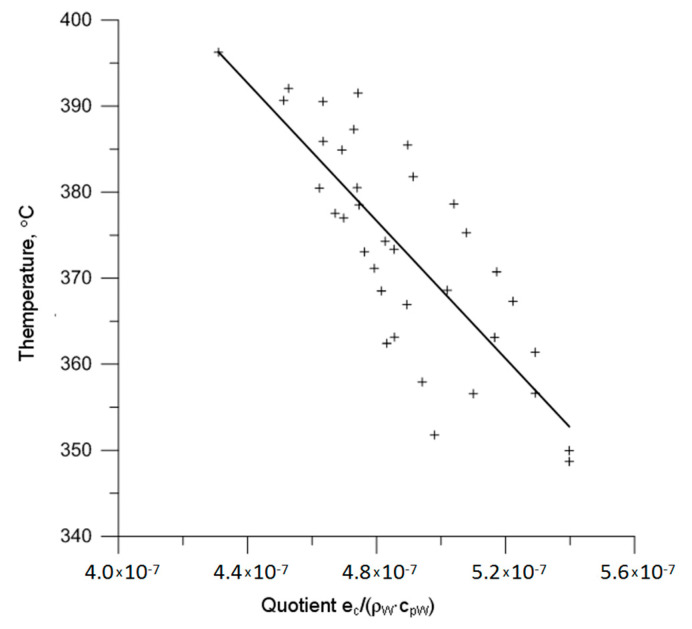
The function graph of changes of the maximum temperature of the chip surface versus the quotient *e_c_*/(*ρ_W_·c_pW_*).

For comparison, Figure 9 shows the changes of the maximum temperature of the chip surface of the test steel AISI 321 versus the quotient *e_c_*/(*ρ_W_·c_pW_*). This is the basis whereon Equation (1) was determined and referenced in the above considerations.

**Figure 10:**
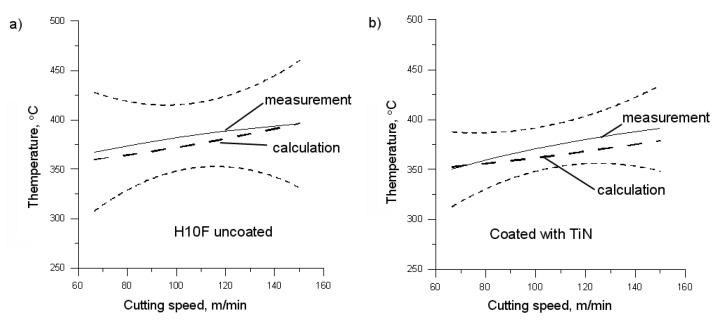

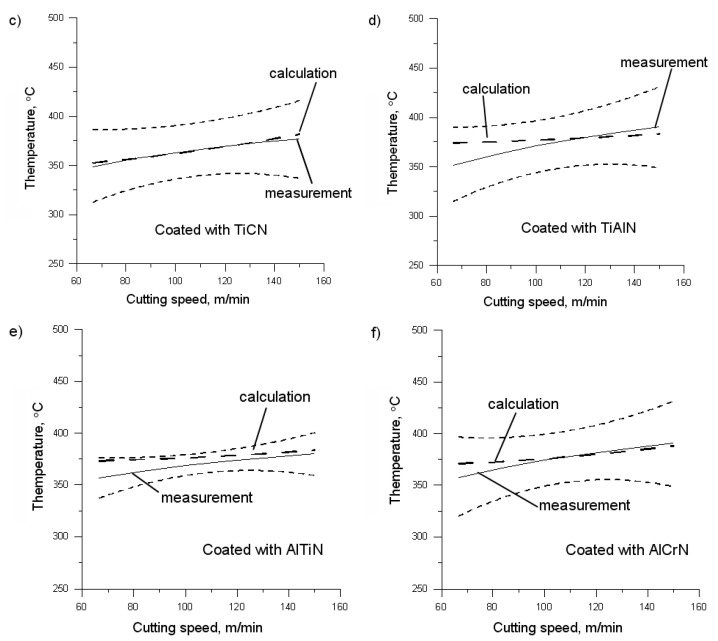
The measured and calculated maximum temperature of the chip surface for the H10F insert uncoated (**a**), coated with TiN (**b**), TiCN (**c**), TiAlN (**d**), AlTiN (**e**), and AlCrN (**f**).

Figure 10 depicts a graphic presentation of the calculation results. The comparison of measured and calculated value of the maximum temperature of the chip surface proves a good matching of the obtained results. The greatest differences were recorded for coatings TiAlN, AlTiN, and AlCrN, especially at a slow cutting speed. The measured and calculated value of the temperature of the chip surface differed in the range of 3.7 to 6.4%, depending on the type of coating. The reason for these discrepancies can be traced to different thermophysical properties of the coatings in this group. The thermal conduction of these materials, especially in the lower temperature, is much less than that of the coatings based on TiN, TiCN, or the carbide H10F itself [18,19,20,21].

In summarising the above considerations, it should be stated that the difference between the course of the maximum temperature of the top surface of the chip from measurements and from the calculations according to Formula (2) does not exceed 6.4%. Therefore, it can be concluded that the proposed Formula (2) for calculating the maximum temperature of the chip top surface meets its purpose and it can be used to analyse the phenomenon of heat distribution in the cutting zone or to validate the results of numerical calculations.

## 4. Conclusions

For researchers that are involved in machining analysis, accurate information regarding the temperature distribution in the cutting zone is important, as it allows for an analysis of the heat distribution in the cutting zone or the validation of calculations in numerical modelling. This paper presents a methodology for the approximation determination of the maximum temperature of the top surface of the chip that brings us closer to this knowledge. All of the tests and analyses presented were carried out on AI-SI 321 austenitic steel, taking this material as a reference. Although the experiments were only carried out for this one steel, the results can be used to determine the chip temperature for other materials, as the physical phenomena occurring during the turning process are very similar in nature. The work undertaken has made it possible to develop a formula for calculating the maximum temperature of the top surface of the chip, *t_Wmax_*, for different machined materials. Verification calculations have shown that calculations of the maximum temperature of the top surface of the chip using the developed formula show good agreement with the experimental results. Therefore, it can be considered that the formula for calculating *t_Wmax_* can find practical application. In the future, it can be successfully used to estimate the heat distribution in the cutting zone.

## Figures and Tables

**Table 1:** Comparison of the protective coatings selected for testing.

Designation of Coating According to Oerlikon Balzers	Type of Coating	Structure of Coating
BALINIT A	TiN	Monolayer
BALINIT B	TiCN	Graded
BALINIT FUTURA NANO	TiAlN	Nanostructured
BALINIT X.CEED	AlTiN	Monolayer
BALINIT HELICA	AlCrN	Multilayer

## Data Availability

The data presented in this study are available on request from the corresponding author.
